# Homologous Recombination in Thyroid Tumor Samples

**DOI:** 10.3390/ijms26199716

**Published:** 2025-10-06

**Authors:** Liudmila V. Spirina, Matvey M. Tsyganov, Svetlana Yu. Chizhevskaya, Natalia V. Tarasenko, Veronika A. Bogdanova

**Affiliations:** 1 Biochemistry and Molecular Biology Division, Siberian State Medical University, 2 Moskovsky Trakt, Tomsk 634050, Russia; tsyganovmm@ya.ru (M.M.T.); sch@oncology.tomsk.ru (S.Y.C.); natvt2003@mail.ru (N.V.T.); milki.ly@mail.ru (V.A.B.); 2 Cancer Research Institute, Tomsk National Research Medical Center of the Russian Academy of Sciences, 5 Kooperativny Street, Tomsk 634050, Russia

**Keywords:** thyroid carcinoma, homologous recombination genes, *FANCA* gene, *BARD1* mutation

## Abstract

Genomic studies have provided key insights into the molecular pathogenesis of differentiated thyroid carcinoma (DTC), including the role of genes involved in the homologous recombination (HR) related to DNA repair and genomic stability. This research aimed to investigate the genetic landscape of HR genes in thyroid pathology, associated with recurrence risk and clinical prognosis. The study involved six individuals with thyroid conditions, including two patients diagnosed with papillary thyroid carcinoma (PTC) and four individuals with benign thyroid disease. The research material consisted of tumor samples collected during surgical procedures. Protein interactions were analyzed using the STRING database (string-db.org). Homologous recombination genes were sequenced using the HRR Panel vr1.0 on the MiSeq™ Sequencing System. Bioinformatics analysis revealed a relationship between *BRAF* mutations and HR gene defects in PTC. Mutations in *BRCA1*, *BRCA2*, and *FANCA* genes, typically associated with thyroid tumors, were identified in the tissue of papillary thyroid cancer (PTC). A statistically significant correlation was found between the *FANCA* gene mutation (rs7195066) and the recurrent course of the PTC. The preliminary findings suggest a potential role for non-pathogenic *BARD1* mutations in follicular adenoma. No significant association was found between genes involved in homologous recombination repair and the incidence of papillary thyroid carcinoma, suggesting that these genes may not play a major role in the development of this type of thyroid cancer.

## 1. Introduction

Differentiated thyroid cancer (DTC), including papillary and follicular carcinomas, is one of the most curable types of cancer [1]. The overall survival rate for patients with DTC is very high, particularly in the early stages [2]. The 10-year survival rate in patients can exceed 95% for localized disease. However, prognosis can vary depending on several factors, such as age, tumor size, extent of disease, and lymph node involvement [1].

The incidence of differentiated thyroid cancer has risen in many regions worldwide. For example, in the United States, the incidence rate of thyroid cancer has more than doubled over the past few decades due to advancements in detection methods and changes in diagnostic criteria [1,2].

Risk factors for DTC can be divided into endogenous and environmental categories. Endogenous factors include genetic predispositions, race, age, gender, obesity, and type 2 diabetes. Environmental risks include exposure to ionizing radiation from medical treatments or nuclear accidents, iodine deficiency, exposure to endocrine disruptors, living in volcanic regions, pollution, and stress [3].

Thyroid cancer is one of the most common endocrine malignancies, with various subtypes exhibiting distinct genetic alterations. Since 2014, genomic studies of differentiated thyroid carcinoma (DTC) using massively parallel sequencing (MPS) have provided key insights into its molecular pathogenesis. DTC has a low mutational burden, with *BRAF*, *RAS*, and fusion genes being the most frequently altered. Novel driver candidates have also been identified, with variations in frequency across DTC subtypes (classical PTC (papillary thyroid cancer), follicular variant of PTC, and follicular thyroid carcinoma). TERT promoter mutations are particularly important for the progression of DTC. Transcriptomic profiling has further classified DTC subtypes based on gene expression, revealing distinct mutational profiles, signaling pathways, and clinicopathological characteristics [1].

Exposure to ionizing radiation is a well-known risk factor, and genetic variations in genes involved in DNA repair may contribute to this risk [1,2]. Our study highlights the importance of ATM-CHEK2-BRCA1 variants in determining the genetic predisposition to PTC and its clinical manifestations. Germline mutations in these genes can be inherited and are present in all body cells, increasing the risk of developing the disease across multiple tissues. In contrast, somatic mutations occur in specific cells after conception and are not inherited, affecting only the cells in which they arise. This distinction is crucial for understanding the genetic basis of PTC and developing personalized treatment strategies. A study by Xu L. et al. (2011) found that *BRCA1* mutations were present in a significant proportion of thyroid cancer cases, suggesting a potential role in disease pathogenesis [3]. Germline mutations in the *FANCA* gene significantly increase the risk of papillary thyroid carcinoma. This emphasizes the critical role of DNA repair in carcinogenesis and enhances our understanding of thyroid cancer etiology. Identifying the *FANCA* gene as a susceptibility factor underscores the importance of genomic stability in preventing neoplastic transformation within the thyroid gland. Further research could lead to targeted therapies and improved risk assessment for individuals carrying *FANCA* mutations [4,5].

Extensive scientific research has clarified the critical role of mutations in homologous recombination (HR) genes in the pathogenesis of distinct subtypes of thyroid carcinoma. Specifically, follicular thyroid carcinoma (FTC) and anaplastic thyroid carcinoma (ATC) exhibit a significantly higher prevalence of these genetic alterations. A comprehensive study by Nagano et al. (2025) has demonstrated that *BRCA2* and *PIK3CA* mutations are positively correlated with a more favorable prognostic profile in ATC patients [6]. On the contrary, mutations in the *STK11* gene have been definitely linked to poorer clinical outcomes [6].

Functional and network analyses have revealed that these DNA repair proteins interact both within and across different pathways, with MAPK activation being common in tumor progression [7,8]. The geographical distribution of mutations in HR genes is particularly noteworthy [9]. Although the Polish population shares genetic similarities with other European populations, there are significant differences in variant frequencies that contribute to disease development and progression, particularly in genes such as the *RET*, *CHEK2*, *BRCA1*, *SLC26A4*, or *TERT* genes. Further studies are necessary to identify genomic variants directly associated with DTC [10,11,12,13].

The *BRAF* gene encodes a protein that is part of the RAS/RAF/MEK/ERK signaling pathway, which regulates cell growth and proliferation [10]. Mutations in the BRAF gene, particularly the V600E mutation, are common in various cancers, including papillary thyroid carcinoma (PTC) [13]. These mutations lead to the constitutive activation of the MAPK signaling pathway, promoting cell proliferation and survival [11].

Genes involved in HR, such as *BRCA1* and *BRCA2*, play a crucial role in DNA repair and maintaining genomic stability [13]. Defects in these genes can impair DNA repair mechanisms and increase genomic instability, contributing to tumorigenesis [10]. The relationship between *BRAF* mutations and defects in HR genes in PTC is complex [13,14]. However, it is clear that both *BRAF* mutations and HR deficiency can contribute to the PTC progression through different mechanisms [6,7]. Understanding how *BRAF* mutations interact with HR status in PTC could pave the way for more effective treatment strategies [15]. For example, targeting the MAPK pathway with BRAF or MEK inhibitors may be more effective for PTC cases with BRAF mutations and proficient homologous recombination. Conversely, therapies that exploit HR defects, such as PARP inhibitors, may prove more effective for PTC cases with homologous recombination deficiency [16].

Comprehensive sequencing of HR genes in thyroid cancer tissues has provided crucial insights into the PTC genetic landscape, focusing on the molecular mechanisms underlying HR deficiency and its implications for clinical prognosis and recurrence risk. The aim of the research was to investigate the genetic landscape of HR genes in thyroid pathology associated with the recurrence risk and clinical prognosis.

## 2. Results

### 2.1. Bioinformatics Analysis of Genes Associated with PTC

Furthermore, bioinformatics tools were used to analyze the effects of *BRAF* mutations, associated with HR deficiency, on protein–protein interactions. Table 1 summarizes the data, highlighting the gene defects identified in PTC. It verifies the RET fusion with the *CCDC6* gene, which is typical for PTC. The other essential mechanisms are found to be associated with the defects in transcriptional activity regulation, post-translational modifications of proteins, and signal transduction pathways: achaete-scute homolog 1 (*ASCL1)*, *TCIM* gene (also known as TC1, TC-1, C8orf4), N-alpha-acetyltransferase 15 (*NAA15*), ATP binding cassette subfamily A member 3 (*ABCA3)*, protospacer adjacent motif (*PAM*) (a DNA sequence that plays an important role in the CRISPR-Cas system), ghrelin O-acyltransferase (*MBOAT4*), *CALCA* (calcitonin-related polypeptide alpha), *CACNA1H * (encodes a low-threshold potential-dependent calcium channel of the T-type Cav3), *GHRL* (ghrelin/obestatin prepropeptide), GDNF family receptor alpha-1, Ras-responsive element-binding protein 1 (*RREB1*), small nuclear ribonucleoprotein-associated proteins B (*SNRPB*), N-alpha-acetyltransferase 10 (NatA catalytic subunit Naa10 and arrest-defective protein 1 homolog A, *NAA10*), nuclear receptor coactivator 4 (Androgen Receptor Activator), and *TCIM* (C8orf4, TC-1, TC1, regulates the immune response).

Bioinformatics analysis provided valuable insights into the relationship between gene defects in PTC. We identified a set of prospective markers of the genetic and epigenetic regulation of gene and protein translation. The DNA repair defects observed in PTC [11,12,13] are believed to be verified by additional data on somatic mutations in thyroid cancers.

Therefore, the findings suggest a complex picture of PTC progression, involving a large number of genes that encode multiple proteins.

### 2.2. Protein–Protein Interactions in PTC

Protein–protein interactions are crucial for regulating cell growth, proliferation, and DNA repair. It is found that BRAF protein can interact with other proteins in the MAPK pathway, such as MEK and ERK, to regulate cell proliferation and survival. Similarly, proteins involved in HR, such as *BRCA1* and *BRCA2*, can interact with other proteins involved in DNA repair to maintain genomic stability [16]. Additionally, *RET* genes are linked to an increased risk of medullary thyroid cancer, and there may also be a connection to differentiated thyroid cancer, although validation is required. The development of thyroid cancer depends on multiple factors, including environmental influences, lifestyle, and genetic predispositions, even with germline mutations [2].

We analyzed STRING base data on proteins associated with PTC. Germline mutations, occurring in germ cells, can be inherited and influence cancer risk. The effects of these mutations can vary in differentiated thyroid cancers. In the case of PTC, we highlighted the protein–protein interactions between proteins involved in the MAPK pathway and HR pathway affecting the cancer progression [4]. We suggest that defects in HR genes can disrupt interactions between HR proteins and other proteins involved in DNA repair, leading to impaired DNA repair and increased genomic instability. Similarly, *BRAF* mutations can disrupt interactions between BRAF and other proteins in the MAPK pathway, leading to the constitutive activation of the pathway and promoting cell proliferation and survival [17].

**Figure 1:**
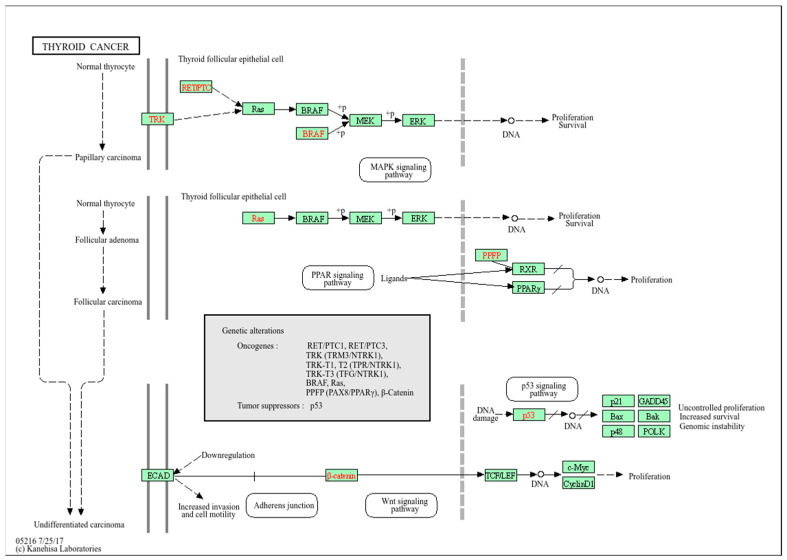
Molecular and genetic factors of thyroid cancer. Note: Approximately 40–50% of papillary thyroid cancers exhibit mutations in *BRAF* or *RAS*, which activate the MAPK signaling pathway. This pathway regulates cell growth, differentiation, and proliferation, making it crucial for cancer development. Activated MAPK signaling promotes cell survival and resistance to apoptosis, thereby facilitating tumor progression. In addition, HR deficiency has been linked to thyroid cancer. These genes are essential for DNA repair and maintaining genomic stability, leading to uncontrolled cell growth and tumor formation.

By understanding how *BRAF* mutations and HR gene defects affect protein–protein interactions, we can gain insights into the mechanisms underlying the development and progression of PTC. This may ultimately contribute to the development of more effective treatment strategies for PTC (https://www.kegg.jp/kegg-bin/show_pathway?hsa05216; accessed on 28 July 2025) (Figure 1).

### 2.3. Sequencing of HR Genes

The study revealed that mutations in *BRCA1*, *BRCA2*, and *FANCA*, characteristic of benign thyroid tumors, were detected in PTC tissues (Table 2). The study noted a relationship between mutations in the *FANCA* gene and the risk of PTC [5]. Furthermore, *BRCA1* rs16941 has shown ambiguous implications regarding its pathogenicity. The literature also suggests a connection between breast tumors and papillary thyroid cancer in women who carry germline mutations in *BRCA1.*

Mutations in *ATR*, *BARD1*, *BLM*, *BRCA2*, *BRIP*, *CHEK1*, *FANCE*, *NBN*, and *RPA1*, which are typically associated with benign neoplasms, were identified in the adenoma tissue (Table 3). Mutations in *ATR* (rs1802904) and *BARD1* (rs2070093) were found in most cases of adenomas. The other mutations were present in a benign variant and were only observed in two patients.

There was ambiguous data regarding the role of *BARD1* rs752628149 and rs757953605 in assessing pathogenicity for one patient with adenoma (see Supplementary Materials). It is known that these changes are often linked to breast cancer. The identified mutations were subsequently analyzed using the ClinVar database (https://clinvarminer.genetics.utah.edu/; accessed on 28 July 2025), and associations were noted with multiple tumors in the presence of *BARD1* mutations with an ambiguous interpretation of pathogenicity [6], as well as for familial forms of breast cancer and hereditary cancer-predisposing syndrome.

### 2.4. Limitations of the Study

#### 2.4.1. Small Sample Size

The study included only six participants, which may not be sufficient to draw generalizable conclusions about the role of homologous recombination genes in thyroid cancer. A larger sample size would provide more robust results and improve the statistical power of the study.

#### 2.4.2. Limited Genetic Analysis

The study focused on a specific set of genes involved in homologous recombination. Other genes and genetic pathways may also play a role in thyroid cancer development and progression, which were not examined in this study.

#### 2.4.3. Single-Center Study

Participants were recruited from a single medical center, which may introduce selection bias and limit the generalizability of the findings to other populations or clinical settings.

#### 2.4.4. Lack of Long-Term Follow-Up

**Lack of long-term follow-up**. The study did not include a long-term follow-up for participants, which would provide valuable insights into clinical outcomes and the prognostic significance of the identified genetic alterations.

#### 2.4.5. Technical Limitations

The sequencing and bioinformatics analysis methods used in the study may have technical limitations that could affect the accuracy and completeness of the genetic data obtained.

#### 2.4.6. Inability to Establish Causality

The observational nature of the study does not allow for establishing causal relationships between the identified genetic alterations and the development of thyroid cancer. Further experimental studies are needed to determine the functional effects of these genetic changes.

## 3. Discussion

PTC is the most common thyroid cancer, with a rapidly increasing incidence. It usually remains confined to the thyroid gland and has an excellent prognosis, with less than 2% mortality at 5 years. However, more than 25% of patients experience recurrence during long-term follow-up [18,19]. This article provides an updated overview of PTC, focusing primarily on the molecular alterations involved and recent investigations into biomarkers.

The study suggests that the risk of adverse outcomes in patients with thyroid cancer is likely associated with certain genetic predictors influencing radiotherapy sensitivity. It found a significant association between the *XRCC3* gene and PTC susceptibility [17]. It highlights the involvement of repair systems, including the genes responsible for HR, which may be linked to patient survival rates. There is insufficient evidence to warrant systematic thyroid screening in *CHEK2* carriers [20].

It has been observed that these changes are more pronounced in individuals diagnosed with cancer compared to healthy controls. This finding aligns with previous research indicating a correlation between specific genetic variations and treatment response. The identified genetic markers could potentially serve as prognostic indicators, enabling personalized therapeutic approaches tailored to individual tumor characteristics.

The study explored the potential genetic factors associated with recurrence risk, specifically focusing on genes involved in HR [1,2,3]. Two patients experienced cancer recurrence but successfully underwent radioiodine therapy, which demonstrated positive therapeutic outcomes; both had mutations in the *FANCA* gene. This finding highlights the potential role of genes in predicting the benefits of anti-cancer therapy [5].

Other patients showed favorable outcomes, and we found *BRCA1* and *BRCA2* mutations in cancers (Table 2). In addition, it has been shown that *BRCA1* (rs16941) has an ambiguous value in assessing pathogenicity.

Preliminary data suggests a potential link between non-pathogenic *BARD1* mutations in follicular adenoma. This finding opens up new avenues for understanding the genetic factors involved in the progression of benign thyroid tumors [6]. BARD1 has been implicated in additional pathways crucial for tumor suppression, independent of the BRCA1 pathway. One such pathway is the TP53-dependent apoptotic signaling cascade [21], suggesting a potential mechanism for anti-cancer defense in benign tumors.

The identification of specific genetic markers could inform genetic counseling for individuals at risk of developing thyroid carcinoma, allowing for more informed decision-making regarding preventive measures and monitoring [8]. The study’s focus on the genes involved in homologous recombination repair and other genetic markers provides new insights into the potential genetic factors contributing to thyroid carcinoma prognosis. The identification of a significant correlation between the *FANCA* gene mutation and thyroid carcinoma represents a novel finding that could open up new areas of research for understanding the genetic features of anti-tumor response [16]. The preliminary data, suggesting a connection between non-pathogenic *BARD*1 mutations and malignant neoplasms in follicular adenoma, offers a fresh perspective on the genetic factors that may protect against the transition of benign tumors to cancer.

## 4. Materials and Methods

### 4.1. Bioinformatic Analysis of Proteins

Protein interactions were analyzed using the STRING database (string-db.org), a comprehensive database for exploring functional protein associations. STRING integrates multiple data sources to provide a holistic view of protein interactions, making it a valuable tool for bioinformatics research.

STRING integrates data from experimental methods such as yeast two-hybrid assays, Co-IP, and mass spectrometry. It also mines text from the literature to identify interactions. STRING draws information from databases like UniProt, IntAct, BioGRID, and others. Additionally, it utilizes computational predictions based on protein structures, evolutionary conservation, and computational algorithms.

STRING enables functional enrichment analysis to identify biological pathways and processes linked to the proteins of interest. It helps to understand their roles in cellular functions. STRING offers interactive network visualizations to explore protein interaction networks, aiding in the analysis of complex protein organization and dynamics. To ensure the reliability of the results, a confidence cutoff of 0.7 was applied. This threshold filters out interactions with lower confidence scores, focusing on the most robust and reliable associations.

### 4.2. Experimental Analysis

The study involved six individuals with thyroid pathology. The clinical details are summarized in Table 4. The study cohort consisted of two patients diagnosed with papillary thyroid carcinoma and four individuals exhibiting benign thyroid pathology.

The entire cohort of patients underwent surgical intervention at the specialized facilities of the Tomsk National Research Medical Center, Cancer Research Institute. The diagnostic process was meticulously executed, with each patient’s condition confirmed through rigorous morphological verification procedures, ensuring the highest level of diagnostic accuracy.

The study was conducted with the approval of the Ethics Committee of the Tomsk National Research Medical Center’s Cancer Research Institute (protocol code 25 dated 17 November 2021). All procedures involving human participants were carried out in accordance with the ethical guidelines outlined in the Helsinki Declaration on Human Rights (1964). Each participant in the study provided a voluntary written informed consent after receiving an explanation of the potential risks and benefits.

#### 4.2.1. Material

The research material consisted of surgical samples from thyroid tumor tissue, as well as samples from benign neoplasms (~30–50 mm^3^). The samples were placed in RNAlater solution (Ambion, Austin, TX, USA). After 24 h incubation at +4 °C, the tumor samples were stored at a temperature of –80 °C for further DNA extraction.

#### 4.2.2. DNA Extraction

DNA was extracted from the tumor samples using a QIAamp DNA Mini Kit (Qiagen, Hilden, Germany) according to the manufacturer’s instructions. DNA concentration was assessed with a fluorometer Qubit 4.0 (Thermo Fisher Scientific, Waltham, MA, USA) and ranged from 20 to 100 ng/mkl. DNA integrity was assessed using capillary electrophoresis on a TapeStation (Agilent Technologies, Santa Clara, CA, USA) device using the Agilent Genomic DNA ScreenTape kit.

#### 4.2.3. Sequencing of Homologous Recombination Genes

The homologous recombination genes were sequenced using the HRR Panel v1.0 (Nanodigmbio, Nanjing, China) on the MiSeq™ Sequencing System (Illumina, San Diego, CA, USA) through bridge amplification. Libraries were created from tumor DNA and patient’s blood DNA (as a control for germinal mutations) using the NadPrep EZ DNA Library Preparation Kit (Nanodigmbio, China). Previously, the double-stranded DNA sequence was fragmented into sections 200–300 bp in size. Then, partially complementary oligonucleotides (adapters) were ligated to both ends of the fragmented DNA using DNA ligase. The resulting fragments were amplified using PCR, forming a set of fragments with adapters at both ends. The resulting libraries were immobilized on prepared flow cells inside the sequencer, where a cyclic sequencing process was carried out through bridge PCR. After amplification, excess copies were removed using a restriction enzyme, leaving behind unidirectional DNA fragments on the cell.

#### 4.2.4. Bioinformatics Analysis in Sequencing

Data processing was performed using a pipeline that includes tools such as BWA, samtools, fastp, and GATK. The genetic variants found in the thyroid tumor tissue samples were annotated using the GATK tool. In particular, the following steps were performed:

- Reading the alignment to the reference genome was performed using BWA-MEM (v0.7.17) with the following parameters: -t 8-M -R ‘@RG\tID:sample1\tSM:sample1\tPL:ILLUMINA’.
- Filtering and sorting of BAM files were performed using samtools (v1.9) with the following parameters: view -b -q 20 -F 4, sort, index.
- Trimming and filtering of reads were performed by fastp (v0.23.2) with the parameters: -q 20 -u 30—length_required 30—detect_adapter_for_pe -w 8.
- Calling of variants was performed using GATK HaplotypeCaller (v3.3) with the parameters:—min-base-quality-score 20—standard-min-confidence-threshold-for-calling 30—native-pair-hmm-threads 8.

To assess the clinical relevance of the identified variants, databases such as OMIM, ClinVar, Human Gene Mutation Database, Human Genome Variation Society, and BRCA Exchange were used, and the literature data was reviewed.

Germline mutation analysis has not been validated.

The minor allele frequency (MAF) threshold for filtering was 1% (MAF < 0.01) to identify rare variants. The pathogenicity of variants was classified according to ACMG recommendations and included the following categories: pathogenic, likely pathogenic, variant of uncertain significance (VUS), likely benign, and benign. Any conflicts in annotations were resolved by integrating data from clinical databases (ClinVar, etc.), functional studies, and expert assessment using standardized ACMG criteria.

## 5. Conclusions

The study examined the genetic factors in thyroid carcinoma, focusing on homologous recombination repair genes and other markers. However, no significant association was found between these genes and papillary thyroid carcinoma, suggesting that they may not play a critical role in its development. Our findings provide valuable insights into the complex relationship between genetics and clinical outcomes in PTC. These data emphasize the importance of incorporating genomic profiling into routine clinical practice to enhance precision medicine efforts aimed at reducing the morbidity and mortality associated with this disease.

A significant correlation was observed between *FANCA* mutation (rs7195066*) and thyroid carcinoma, highlighting its potential role in the disease and warranting further research. Additionally, the preliminary data suggested a connection between non-pathogenic *BARD1* mutations and the progression of follicular adenoma to malignant neoplasms, offering new insights into benign-to-malignant transformation.

Identifying genetic markers could lead to early detection tools, prevention strategies, and personalized treatments, improving patient outcomes. The findings of the study regarding *FANCA* and *BARD1* mutations provide novel insights into the genetic basis of thyroid cancer.

## Figures and Tables

**Table 1:** List of genes associated with PTC.

Name	Source	Evidence	Confidence
*CCDC6*	UniProtKB-RC	CURATED	★★★★☆
*ASCL1*	UniProtKB-RC	CURATED	★★★★☆
*NAA15*	UniProtKB-RC	CURATED	★★★★☆
*ABCA3*	UniProtKB-RC	CURATED	★★★★☆
*PAM*	UniProtKB-RC	CURATED	★★★★☆
*MBOAT4*	UniProtKB-RC	CURATED	★★★★☆
*TCIM*	UniProtKB-RC	CURATED	★★★★☆
*CALCA*	UniProtKB-RC	CURATED	★★★★☆
*CACNA1H*	UniProtKB-RC	CURATED	★★★★☆
*GHRL*	UniProtKB-RC	CURATED	★★★★☆
*GFRA1*	UniProtKB-RC	CURATED	★★★★☆
*RREB1*	UniProtKB-RC	CURATED	★★★★☆
*SNRPB*	UniProtKB-RC	CURATED	★★★★☆
*NAA10*	UniProtKB-RC	CURATED	★★★★☆
*NCOA4*	UniProtKB-RC	CURATED	★★★★☆
*TCIM*	UniProtKB-RC	CURATED	★★★★☆
*RET*	UniProtKB-RC	CURATED	★★★★☆

Note: The tissue associations are based on manually curated knowledge in UniProtKB and automatic text mining of the biomedical literature, which has not been manually verified. The confidence level for each association is indicated by stars, with ★★★★★ representing the highest confidence and ★☆☆☆☆ the lowest. Each tissue–gene association is based on a text-mining score, which is proportional to (1) the absolute number of co-mentions and (2) the ratio of observed to expected co-mentions (i.e., the enrichment). These scores are normalized to z-scores by comparing them to a random background. This is represented by stars, with each star corresponding to two standard deviations above the mean of the background distribution.

**Table 2:** List of gene mutations detected in the tissue of patients with papillary thyroid cancer.

Gene	Mutation Class	Mutation	avsnp150	Type
*BRCA1*	Synonymous SNV	c.4308T>A (p.Ser1436)	rs1060915	Benign
*BRCA1*	Nonsynonymous SNV	c.3113A>C (p.Glu1038Ala)	rs16941	Conflicting classifications of pathogenicity
*BRCA2*	Synonymous SNV	c.4563A>G (p.Leu1521)	rs206075	Benign/Little Clinical Significance
*BRCA2*	Nonsynonymous SNV	c.7397= (p.Val2466)	rs169547	Benign
*FANCA*	Nonsynonymous SNV	c.2426G>A (p.Gly809Asp)	rs7195066	Benign

**Table 3:** List of gene mutations detected in the tissue of patients with thyroid adenoma.

Gene	Mutation Class	Mutation	avsnp150	Type
*ATR*	Synonymous SNV	c.7875G>A (p.Gln2625=)	rs1802904	Benign/Likely_benign
*ATR*	Synonymous SNV	c.5208T>C (p.Tyr1736)	rs2227931	Benign/Likely_benign
*BARD1*	Synonymous SNV	c.1518T>C	rs2070093	Benign
*BARD1*	Nonsynonymous SNV	c.1519G>T	rs2070094	Benign/Likely_benign
*BLM*	Synonymous SNV	c.3102G>A (p.Thr1034)	rs2227933	Benign
*BLM*	Synonymous SNV	c.3531C>A (p.Ala1177)	rs2227934	Benign
*BRCA2*	Synonymous SNV	c.4563A>G (p.Leu1521)	rs206075	Benign
*BRIP1*	Synonymous SNV	c.3411T>C (p.Tyr1137)	rs4986763	Benign
*CHEK1*	Nonsynonymous SNV	c.1411A>G (p.Ile471Val)	rs506504	Benign
*FANCE*	Synonymous SNV	c.387A>T (p.Pro129=)	rs4713867	Uncertain significance
*NBN*	Synonymous SNV	c.2016A>C (p.Pro672=)	rs1061302	Benign
*RPA1*	Synonymous SNV	c.12A>G	rs5030749	Benign

**Table 4:** Clinical data of patients included in the study.

Code	Gender	Diagnosis	Age	Recurrence	Prognosis	Patients Observation Time
MTEX71	Female	Diffuse toxic goiter	41	No	Alive	24
BIOQ72	Female	Papillary thyroid cancer T1N0M0	36	Yes	Alive	36
DNAR73	Female	Papillary thyroid cancer T2N0M0	74	Yes	Alive	36
CELL74	Female	Nodular colloidal macro-microfollicular goiter, with patches of fibrosis, hyalinosis	70	No	Alive	18
XRAY75	Female	Macro-microfollicular colloidal goiter with focal regressive changes and focal hyperplasia of the thyroid epithelium.	66	No	Alive	12
GENF76	Female	Follicular adenomas of the thyroid gland on the background of nodular colloidal goiter	31	No	Alive	10

Note: Two patients experienced a recurrence of cancer but successfully underwent radioiodine therapy, which yielded positive therapeutic outcomes.

## Data Availability

The original contributions presented in this study are included in the article/Supplementary Materials. Further inquiries can be directed to the corresponding author.

## Supplementary Materials

The following supporting information can be downloaded at https://www.mdpi.com/article/10.3390/ijms26199716/s1.

## Abbreviations

The following abbreviations are used in this manuscript:

ATC	Anaplastic thyroid carcinoma
DTC	Differentiated thyroid carcinoma
FNMTC	Familial Non Medullary Thyroid Carcinoma
HR	Follicular thyroid carcinoma
MPS	Massively parallel sequencing
PTC	Papillary thyroid cancer
